# Structural defects on converted bismuth oxide nanotubes enable highly active electrocatalysis of carbon dioxide reduction

**DOI:** 10.1038/s41467-019-10819-4

**Published:** 2019-06-26

**Authors:** Qiufang Gong, Pan Ding, Mingquan Xu, Xiaorong Zhu, Maoyu Wang, Jun Deng, Qing Ma, Na Han, Yong Zhu, Jun Lu, Zhenxing Feng, Yafei Li, Wu Zhou, Yanguang Li

**Affiliations:** 1 Institute of Functional Nano & Soft Materials (FUNSOM), Jiangsu Key Laboratory for Carbon-Based Functional Materials and Devices, Soochow University, Suzhou, 215123 China; 2 School of Physical Sciences and CAS Key Laboratory of Vacuum Sciences, University of Chinese Academy of Sciences, Beijing, 100049 China; 3 College of Chemistry and Materials Science, Nanjing Normal University, Nanjing, 210023 China; 4 School of Chemical, Biological, and Environmental Engineering, Oregon State University, Corvallis, OR 97331 USA; 5 DND-CAT, Synchrotron Research Center, Northwestern University, Evanston, IL 60208 USA; 6 Chemical Sciences and Engineering Division, Argonne National Laboratory, Lemont, IL60439 USA

**Keywords:** Electrocatalysis, Electrochemistry

## Abstract

Formic acid (or formate) is suggested to be one of the most economically viable products from electrochemical carbon dioxide reduction. However, its commercial viability hinges on the development of highly active and selective electrocatalysts. Here we report that structural defects have a profound positive impact on the electrocatalytic performance of bismuth. Bismuth oxide double-walled nanotubes with fragmented surface are prepared as a template, and are cathodically converted to defective bismuth nanotubes. This converted electrocatalyst enables carbon dioxide reduction to formate with excellent activity, selectivity and stability. Most significantly, its current density reaches ~288 mA cm^−2^ at −0.61 V versus reversible hydrogen electrode within a flow cell reactor under ambient conditions. Using density functional theory calculations, the excellent activity and selectivity are rationalized as the outcome of abundant defective bismuth sites that stabilize the *OCHO intermediate. Furthermore, this electrocatalyst is coupled with silicon photocathodes and achieves high-performance photoelectrochemical carbon dioxide reduction.

## Abstract (web summary)

Carbon dioxide can be electrochemically reduced to form valuable chemical feedstocks, but efficiency of electrocatalysts should be improved. Here the authors report nanotube-derived bismuth for electrocatalytic reduction of carbon dioxide to formate, with performance that is enhanced by defects.

## Introduction

Electrochemical CO_2_ reduction can potentially enable the direct transformation of renewable electricity to valuable chemical feedstocks or fuels, thereby closing the anthropogenic carbon cycle^1–5^. However, its industrial viability is yet to be proven, and essentially contingent upon the development of efficient electrocatalyst materials for CO_2_ reduction reaction (CO_2_RR)^6–8^. The challenges come from not only the sluggish reaction kinetics owing to the relative chemical stability of CO_2_ molecules, but more seriously the limited selectivity caused by the sophisticated CO_2_RR pathways and competing proton reduction process. A multitude of possible CO_2_RR products have been experimentally identified, ranging from C_1_ products including CO and formic acid (or formate) to multicarbon (C_2+_) products such as ethylene and ethanol^2^. Even though it is intuitively more desirable to steer the selectivity toward C_2+_ products for their higher industrial values^9–12^, recent technoeconomic analysis shows that the production of CO or formic acid is more economically viable^2,13^. They can be further upgraded chemically or electrochemically.

Formic acid (or formate) is an important product from the two-electron reduction of CO_2_. It has been widely explored as the hydrogen storage material, and as the chemical fuel for fuel cells; it is also a key chemical intermediate with great industrial significance^14,15^. The global market size of formic acid was 697,000 ton per year in 2013 and is expected to grow to approximately 1 million tons by 2030^16^. Carbonylation of methanol is currently the most common strategy for the industrial production of formic acid, which, however, is an energy-intensive process. Direct conversion of CO_2_ to formic acid via the mild and energy-efficient electrochemical approach is highly desired^16^. It is predicted in the “Global Roadmap for Implementing CO_2_ Utilization” by the Global CO_2_ Initiative that the global market size of formic acid from CO_2_ reduction can be up to 475,000 ton year^−1^ by 2030 if suitable electrocatalyst materials are developed^16^. To render the production of formic acid economically compelling, it is suggested that the minimum current density (*j*) required is ~200 mA cm^−2^, Faradaic efficiency (FE) >95% and catalyst durability >1000 h under ambient conditions^2,13^. This sets the performance target if we want to bring any electrocatalyst material from benchtop scale science to industrial scale implementation.

Several IIB, IIIA, and IVA metals (i.e., Pb, Sn, Hg, In, and Cd) have been known for their selectivity toward formic acid or formate since the seminal works by Hori^17–19^. Among these metals, Sn attracts the most attention for its low toxicity and cost. However, Sn-based materials generally suffer from unsatisfactory activity (*j* < 20 mA cm^−2^) and formate selectivity (peak selectivity < 90%)^20–23^. Very recently, Bi quickly rises as a highly promising candidate for selective formate production. Our own studies as well as others have demonstrated that the selectivity of nanostructured Bi-based materials can reach ~100% over a broad potential range^24–28^. But even with these state-of-the-art electrocatalysts, the measured current density is still an order of magnitude lower than the minimum industrial requirement mentioned above.

In order to narrow or close the huge gap in current density, we need to rationally engineer electrocatalyst materials for not only enlarged surface areas, but also enhanced site-specific activities. In particular, the introduction of structural disorders or defects sometimes can influence the local electronic states and create under-coordinated sites with unexpectedly high activity. Even though the underlying mechanism is sometimes elusive, this strategy has been commonly used for many catalytic and electrocatalytic applications^29,30^. Unfortunately, when it comes to Bi, the direct preparation of its metallic nanostructures with regulated structural defects is very challenging. This is because Bi has a low-melting point so its nanostructures are physically unstable, and Bi has a high propensity for oxidation upon air exposure so it is also chemically unstable. We reason that an alternative solution is to design defective Bi compounds (e.g., its oxides) as the template and cathodically convert them to metallic Bi nanostructures rich in defects for electrocatalytic CO_2_RR. The structure of the actual working electrocatalyst thereby can be modulated via tailoring that of the starting template. This strategy is conceptually analogous to the preparation of oxide-derived Au, Cu and Ag nanoparticles reported in literature for high-performance CO_2_RR^31–33^.

Here, we develop a facile solution method to prepare defective β-Bi_2_O_3_ double-walled nanotubes (NTs). These oxide nanotubes can be converted to defective Bi nanotubes under the cathodic polarization as evidenced by *operando* X-ray absorption spectroscopy (XAS) measurements. When evaluated for electrochemical CO_2_ reduction, the converted product enables highly active and selective formate production. Our theoretical studies reveal that this excellent activity and selectivity can be attributed to the presence of abundant defective Bi sites that stabilize the *OCHO intermediate.

## Results

### Preparation of bismuth oxide NTs

**Fig. 1:**
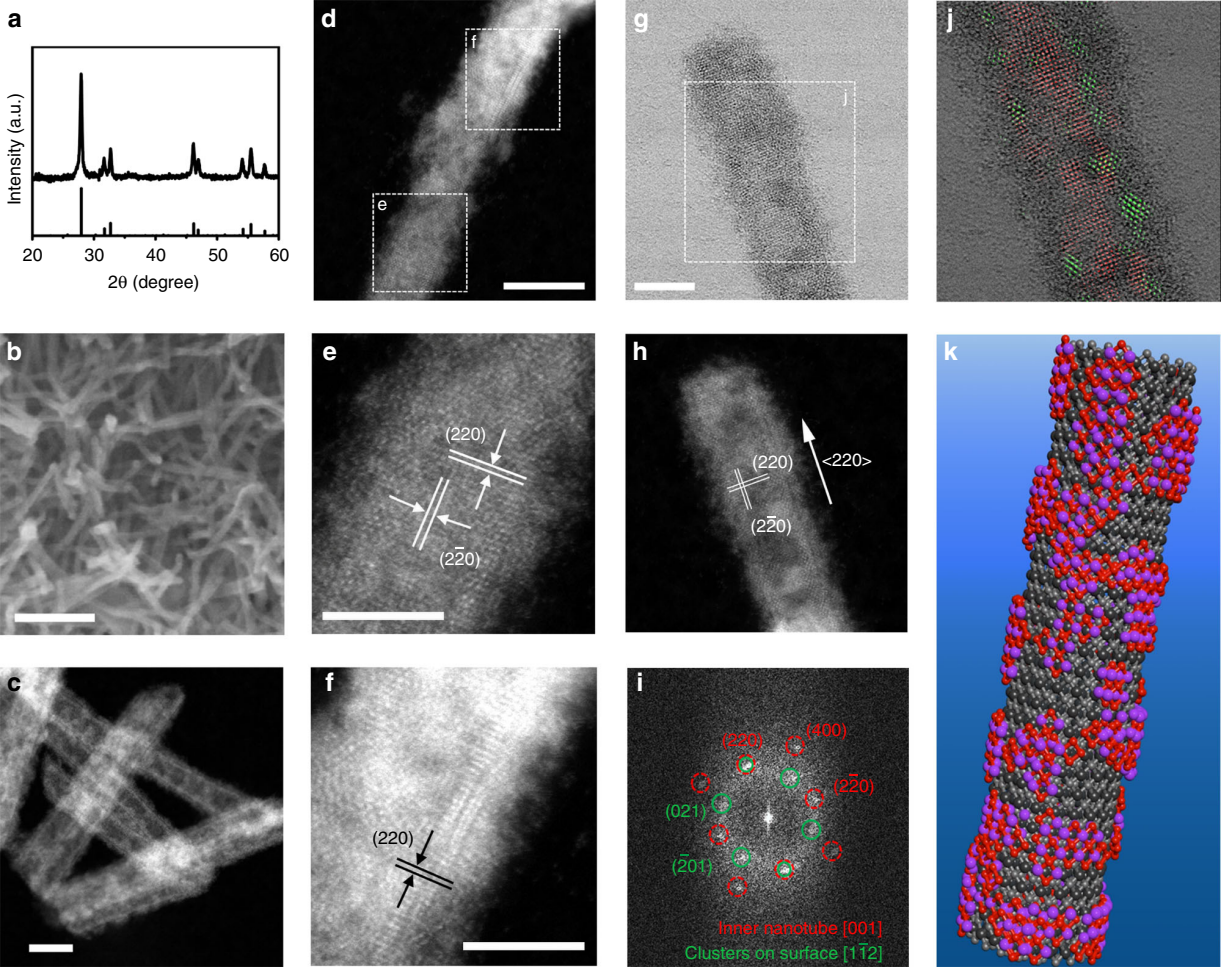
Structural characterizations of bismuth oxide nanotubes. **a** XRD pattern, **b** SEM image, and **c**, **d** STEM-HAADF images of Bi_2_O_3_ NTs. **e**, **f** High-resolution images of the enclosed areas in (**d**). **g**–**i** Simultaneously acquired **g** STEM-BF image, **h** STEM-HAADF image, and **i** corresponding FFT pattern of a Bi_2_O_3_ nanotube (NT). **j** Overlay of the FFT filtered fringes with the original STEM-BF image in (**g**); the fringes in red are constructed by filtering the FFT spots highlighted in red in (**i**), and the green fringes are constructed from the green spots. **k** Schematic illustration of the structure of Bi_2_O_3_ NTs; the black spheres represent the crystalline inner walls, and the red and purple spheres represent the fragmented outer walls. Scale bar, 100 nm (**b**); 10 nm (**c**, **d**); 5 nm (**e**–**g**)

Bi_2_O_3_ NTs were prepared via the controlled hydrolysis of bismuth acetate in ethylene glycol with the presence of poly(vinyl pyrrolidone) (PVP) and a small amount of water (see details in Methods). Key to the synthesis is the introduction of PVP as the structure directing agent. The product is determined to be composed of tetragonal β-Bi_2_O_3_ from its X-ray diffraction (XRD) pattern (Fig. 1a). Scanning electron microscopy (SEM) image shows that it consists of one-dimensional (1D) nanostructures (Fig.1b). In order to resolve the detailed atomic-scale structure of the product, we carried out simultaneous bright field (BF) and high-angle annular dark field (HAADF) imaging on an aberration-corrected scanning transmission electron microscope (STEM). Interestingly, the 1D nanostructures are revealed to have hollow centers, resembling carbon NTs as evident from the STEM-HAADF image in Fig. 1c. These NTs have a length of 30–60 nm and an inner diameter of 4.5 ± 0.2 nm. They are demonstrated to be mostly double-walled, with the longitudinal growth along a 〈220〉 direction of the tetragonal β-Bi_2_O_3_ crystal (Fig. 1d–h). A feature particularly worth noting is that their outer walls are covered with highly defective fragments or clusters. These fragments are presumably resulted from the selective redox etching of NTs by ethylene glycol. Moreover, the crystallographic orientation of Bi_2_O_3_ NTs is confirmed by analyzing the fast Fourier transform pattern of the STEM images (Fig. 1i, j). The main set of spots (in red circles) arise from the crystalline NT projected along the [001] zone axis, while another set of weak spots (in green circles) along the [1–12] zone axis is generated by the small fragments on surface. Electron energy loss spectroscopy analysis of Bi_2_O_3_ NTs shows very weak carbon signal, indicating that there is minute carbon residue on surface (Supplementary Fig. 1). Figure 1k schematically illustrates the structural model of our Bi_2_O_3_ NTs constructed based on the STEM characterization results. Even though the preparation of Bi_2_O_3_ NTs has been sporadically reported^34,35^, our material clearly distinguishes itself from earlier works by its double-walled feature and surface coverage of highly defective fragments. It presents an ideal template for the cathodic conversion to defective metallic Bi nanostructures.

### Electrochemical carbon dioxide reduction performance

For electrochemical measurements, the as-prepared Bi_2_O_3_ NTs were loaded onto carbon fiber paper electrode, and tested inside an H-type electrochemical cell filled with 0.5 M KHCO_3_ using the standard three-electrode configuration (see details in Methods). Upon the initial negative sweep, a pronounced cathodic wave is observed between −0.3 and 0.2 V (versus reversible hydrogen electrode (RHE), the same hereafter) prior to the reduction of water or CO_2_ (Supplementary Fig. 2). This cathodic wave is attributed to the electrochemical reduction of Bi_2_O_3_ to metallic Bi^25^, as supported by the XRD analysis of the reduced electrode showing only the diffraction peaks of rhombohedral Bi (Supplementary Fig. 3a). Remarkably, SEM and low-magnification TEM studies reveal that the 1D tubular morphology with highly fragmented outer surface is largely preserved even after the reduction (Supplementary Fig. 3b–d). Atomic resolution imaging of the reduced material was attempted but unsuccessful since Bi was very sensitive to both oxidation and electron beam irradiation damage. Based on the above characterizations, it can be adequately inferred that metallic Bi is the active component responsible for any subsequent cathodic reaction, even though the possibility of minor oxygen residues on surface cannot be completely excluded. This working electrocatalyst is named as NT-derived Bi (NTD-Bi) to reflect its history and chemical nature.

**Fig. 2:**
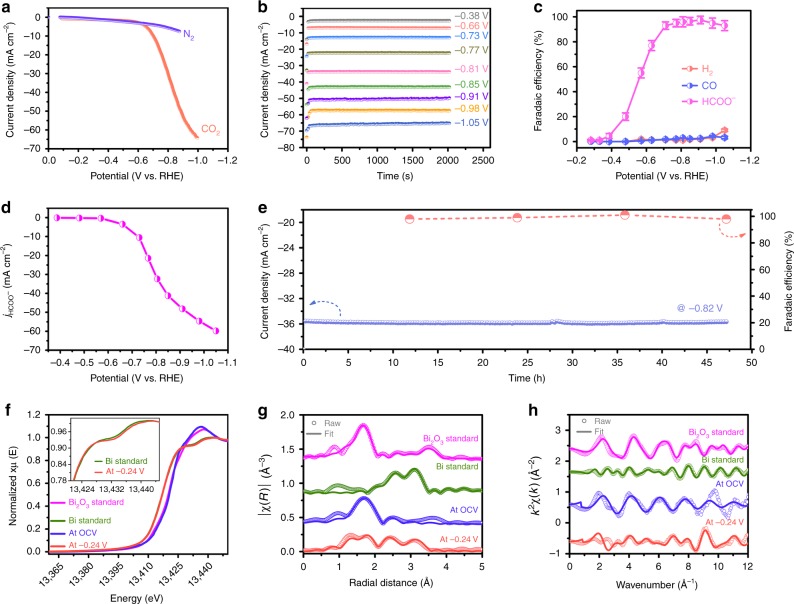
Electrochemical carbon dioxide reduction measurements in the H-type cell. **a** Polarization curves of nanotube-derived Bi (NTD-Bi) in CO_2_ or N_2_-saturated electrolytes. **b** Chronoamperometric responses in CO_2_-saturated electrolyte at different potentials as indicated. **c** Potential-dependent Faradaic efficiencies of HCOO^−^, CO, and H_2_. **d** Formate partial current density derived from (**b**) and (**c**). **e** Long-term amperometric stability and corresponding selectivity change at −0.82 V. **f** *Operando* Bi L-edge XANES spectra of Bi_2_O_3_ nanotubes (NTs) at OCV and NTD-Bi at −0.24 V in comparison with Bi or Bi_2_O_3_ standards; inset plot is the partially enlarged spectra. **g**, **h** Fitting results of the *operando* EXAFS spectra to (**g**) R space and (**h**) K space

Once cathodically converted, NTD-Bi can enable hydrogen evolution reaction (HER) in N_2_-saturated electrolyte or CO_2_RR in CO_2_−saturated electrolyte at less than −0.3 V. Its CO_2_RR polarization curve abruptly takes off at ~−0.6 V, increases sharply and reaches 30 mA cm^−2^ at −0.8 V and 64 mA cm^−2^ at −1.0 V (Fig. 2a). By contrast, the HER polarization curve evolves more mildly and only delivers 5.6 mA cm^−2^ at −0.8 V. Such a stark difference immediately evidences that NTD-Bi strongly favors CO_2_RR over HER. To quantitatively determine the CO_2_ reduction products, NTD-Bi was biased at several selected potentials between −0.28 V and −1.05 V (Fig. 2b). In agreement with previous studies^25–28^, formate is found to be the predominant product from CO_2_ reduction. It is first reliably detected at as early as −0.38 V. The initial FE is calculated to be 4.4%, which then rapidly rises to 93% at −0.7 V, and maintains >93% between approximately −1.0 and −0.7 V (Fig. 2c). In addition to formate, minor amounts (<3%) of CO and H_2_ are also measured. Furthermore, the formate partial current density is derived. It attains an unprecedented value of 60 mA cm^−2^ at −1.05 V (Fig. 2d). As far as we are aware, the combination of great formate selectivity over a broad potential window and great current density is far superior to any other known formate-producing CO_2_RR electrocatalysts (including Sn, In, and N-doped carbon), whose peak formate selectivity (<90%) and peak current density (<20 mA cm^−2^) are generally limited and attained only at high overpotentials (*η* *>* 0.8 V)^36–44^. It is also considerably improved over all previous Bi-based materials^25–28^, in particular on the current density, thereby unambiguously underlining the unique advantage of our NTD-Bi with abundant structural defects.

In addition to its excellent activity and selectivity, NTD-Bi also exhibits satisfactory long-term durability. Bulk electrolysis was performed at −0.82 V for 48 h. The total current density of NTD-Bi stabilizes at ~36 mA cm^−2^ during the course of evaluation (Fig. 2e). Its formate FE was periodically determined by extracting a small volume of electrolyte without interrupting the electrolysis every 12 h. The value is found to be highly consistent and within the range of ~98–100%.

### *Operando* XAS measurements

In order to probe the oxidization state and local structure of Bi during CO_2_RR, we carried out *operando* XAS measurements at the Bi L-edge (see details in Methods). During the measurements, the working electrode was biased at a few selected potentials from the open circuit voltage (OCV) down to −0.78 V. Each potential was held for at least 20 min prior to XAS measurements to ensure that the steady state was reached, and was further kept for at least 1 h for data collection. As shown in Fig. 2f, the Bi edge of the X-ray absorption near edge structure (XANES) at the OCV condition aligns well with that of the Bi_2_O_3_ reference. When the potential is decreased to −0.24 V, the characteristic of Bi(III) edge at 13.423 keV gradually shifts to a lower energy of 13.417 keV that aligns with the XANES edge of Bi metal foil. Further reduction of the working potential results in no additional shift of the XANES edge. Their corresponding XANES spectra are omitted from Fig. 2f for the sake of clarity. Above observation confirms that Bi_2_O_3_ NTs are reduced to metallic Bi prior to CO_2_RR.

Furthermore, we conducted model-based analysis to quantify the *operando* extended X-ray absorption fine structure (EXAFS) results. The models were a combination of scattering paths from Bi_2_O_3_, Bi metal, and the theoretically calculated Bi metal with different absorbed ligands (e.g., *OCHO) on surface. The data and fitted spectra are shown in Fig. 2g, h and the fitting parameters are listed in Supplementary Table 1. It is noted that the Bi–O coordination number (CN) of Bi_2_O_3_ NTs at OCV is calculated to be 3.5 ± 0.6, which is markedly smaller compared with the expected value for the bulk material (CN = 5). This under-coordination can be rationalized by its highly defective nature, in good agreement with our STEM characterization results of Bi_2_O_3_ NTs. At −0.24 V and beyond, Bi_2_O_3_ NTs are reduced to NTD-Bi. The presence of the Bi–O scattering paths from our EXAFS fitting can be reasonably assigned to chemisorbed *OH and *OCHO (intermediate to formate) species (Fig. 2g). This is similar to previous observations on the chemisorbed intermediates such as *OH and *H on Pt surface during electrochemical oxygen reactions^45,46^. Note that the XANES spectrum of NTD-Bi at −0.24 V deviates slightly from that of the Bi metal at the energy around 13435 eV (the inset of Fig. 2f), which is also similar to the change in Pt L-edge XANES when *OH and *H are chemisorbed on Pt metal surface^45–47^. More importantly, the CN of Bi-Bi is determined to be 2.6 ± 1.8 at –0.24 V, significantly smaller than that of Bi metal foil (CN = 6), indicating the existence of many low-coordination sites in NTD-Bi. Even though we are unable to pinpoint the exact atomic configurations of these low-coordination sites, our EXAFS results here provide solid evidence to the presence of abundant defects or vacancies in our material under the actual working condition.

### Flow cell assessments

**Fig. 3:**
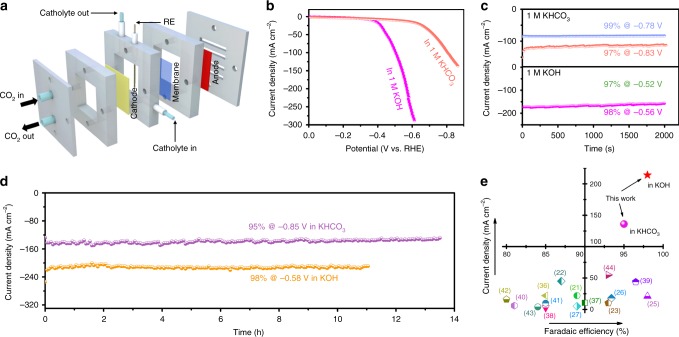
Electrochemical carbon dioxide reduction measurements in the flow cell. **a** Schematic illustration of the flow cell configuration. **b** Polarization curves of nanotube-derived Bi (NTD-Bi) in 1 M KHCO_3_ or 1 M KOH. **c** Chronoamperometric responses at a few different potentials in the two electrolytes. **d** Long-term amperometric stability in the two electrolytes. **e** Comparison of our results with previous data in terms of current density and Faradaic efficiency; corresponding reference numbers are included in brackets

To demonstrate the commercial viability of any formate-producing CO_2_RR electrocatalyst, the minimum current density required is estimated to be ~200 mA cm^−2^. Such a large current density is difficult to attain using conventional working electrode and electrochemical cell setup since CO_2_ has limited solubility in aqueous electrolyte^10^. To this end, we deposited NTD-Bi on a gas diffusion layer (GDL) and assessed its performance using a flow cell reactor (Fig. 3a and Supplementary Fig. 4) (see details in Methods). Within this cell configuration, CO_2_ can be directly supplied to the cathode, quickly diffuse to and react at the solid–liquid–gas triple phase boundaries, thereby lifting the limitation on its electrolyte solubility^10,48^. A peristaltic pump was used in our experiment to force the catholyte circulation so as to dilute the formate accumulation and buffer the electrolyte pH change.

We first tested the flow cell with 1 M KHCO_3_. The CO_2_RR polarization curve of NTD-Bi is observed to have an onset potential at ~−0.6 V (Fig. 3b), which is similar to the value measured from the standard H-type cell. The advantage of using flow cells over conventional cells is clearly manifested by the significantly larger current density. For instance, the former delivers 136 mA cm^−2^ at −0.86 V, while the latter delivers 44 mA cm^−2^ under the same potential. This more than twofold gain in the current density results from the rapid CO_2_ diffusion through GDL. FE analysis unveils that the average selectivity for formate is 99% at −0.78 V and 97% at −0.83 V (Fig. 3c), and hence supports that the large cathodic current density is mostly contributed by CO_2_ reduction to formate.

Another advantage with flow cells is that they permit the use of alkaline electrolytes for CO_2_RR^10^. It is suggested that alkaline electrolytes can suppress the competing HER, and lower the CO_2_RR activation energy barrier on some CO_2_RR electrocatalysts (e.g., Cu)^10^. In addition, alkaline electrolytes promotes the counter reaction, oxygen evolution reaction (OER), so they can improve the overall energy conversion efficiency in actual CO_2_-splitting electrolyzers. When our flow cell is tested with 1 M KOH, the CO_2_RR onset potential is reduced to ~ −0.3 V (Fig. 3b). It is the most incredible that the current density reaches 288 mA cm^−2^ at −0.61 V, which clearly exceeds the commercialization requirement. The formate selectivity in 1 M KOH is also satisfactory, and measured to be 97% at −0.52 V and 98% at −0.56 V (Fig. 3c).

Importantly, the durability of our NTD-Bi is not comprised under large current density. It is shown to sustain ~140 mA cm^−2^ at −0.85 V in 1 M KHCO_3_, and ~210 mA cm^−2^ at −0.58 V in 1 M KOH for 11–13 h (Fig. 3d). The average formate FE is calculated to 95–98%. After the 11 h evaluation in 1 M KOH, the total charge passed is equivalent to the reduction of ~1.0 L of CO_2_ to ~43 mmol of formate. It should be noted that this duration is far from the possible lifetime of our electrocatalyst, but instead limited by our non-optimal flow cell design. The cathode GDL is usually flooded with the electrolyte after continuous operation for >12 h. We are currently working to alleviate or solve this flooding issue. In Fig. 3e, we compare our measured current density and FE with a few best results from recent formate-producing electrocatalysts. Our NTD-Bi clearly outperforms all of them.

### Theoretical simulations

**Fig. 4:**
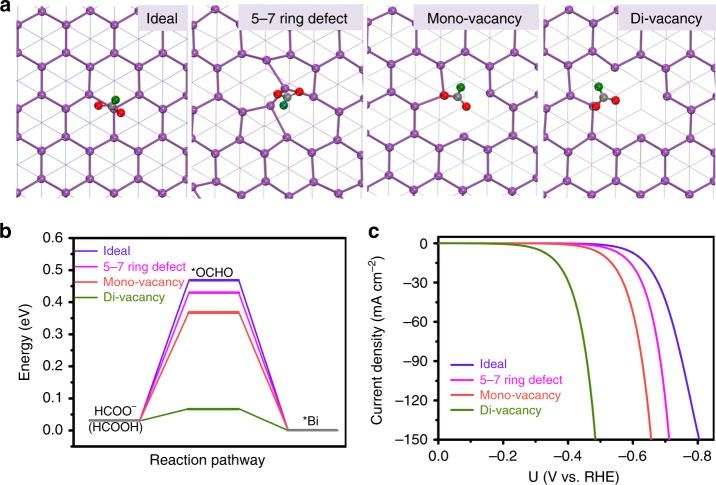
Theoretical calculations of reaction pathway on ideal and defective Bi(001) surfaces. **a** Optimized geometric structures of *OCHO adsorbed on ideal and three defective Bi(001) surfaces as indicated; the pink, gray, red, and green spheres represent Bi, C, O, and H atoms, respectively. **b** Free-energy profiles for formate production on ideal and defective surfaces. **c** Corresponding simulated CO_2_RR polarization curves

Above electrochemical studies establish the extraordinary activity and selectivity as well as the impressive durability of our NTD-Bi electrochemically converted from Bi_2_O_3_ NTs. We believe that it is owing to the abundant structural defects on surface. In order to gain more insights into the possible impact of defects on the CO_2_RR activity and selectivity, density functional theory (DFT) calculations were carried out to simulate and compare the CO_2_RR pathway on ideal and defective Bi surfaces (see details in Methods). Since Bi has a layered structure analogous to graphene and black phosphorus, it became very natural for us to focus on its (001) plane as the most representative surface. The (001) plane is the most stable low index plane and natural cleavage plane in Bi single crystals^49^. We built a trilayer model of rhombohedral Bi(001) in a 3 × 3 supercell, and evaluated the influences of the most common types of structural defects, namely 5–7 ring defect, mono-vacancy and di-vacancy. Figure 4a depicts the optimized adsorption geometry of *OCHO (intermediate to formate) on the ideal and defective surfaces. Their energy profiles are summarized in Fig. 4b. In general, Bi is known to significantly stabilize *OCHO relative to *COOH (intermediate to CO) or *H (intermediate to H_2_). On the ideal Bi(001) surface, the formation of *OCHO is calculated to have a ΔG of +0.47 eV, whereas the ΔG for the formation of *COOH and *H is +1.2 and +0.97 eV, respectively. At the presence of surface 5–7 ring defect or mono-vacancy, ΔG for the formation of *OCHO is lowered to +0.43 or +0.37 eV, respectively (Fig. 4b). Most strikingly, the value is further lowered to +0.07 eV at the presence of di-vacancy. These results indicate that the presence of structural defects can greatly stabilize *OCHO. Similar conclusion can be drawn when the simulation is done on other planes (Supplementary Fig. 5). Even though the adsorption of *COOH and *H is also likewise enhanced on defective Bi surfaces, their formations are still thermodynamically unfavorable compared to *OCHO (Supplementary Fig. 6).

As a step further, we simulated the CO_2_RR polarization curves on these surfaces in CO_2_-saturated 0.5 M KHCO_3_ (pH = 7.2) using the mean-field kinetic model^50,51^. The difference is revealed to be strikingly large (Fig. 4c). At −0.6 V, the current density on ideal surface is only 7 mA cm^−2^, while it can be boosted to 17, 51, and 304 mA cm^−2^ in the presence of 5–7 ring defect, mono-vacancy and di-vacancy, respectively. Because of the simplified theoretical model used and many approximations made, it is not surprising that the simulated polarization curves do not faithfully reflect the experimental data. However, our study firmly captures the profound positive impact of structural defects on the CO_2_RR activity.

### Photoelectrochemical carbon dioxide reduction

**Fig. 5:**
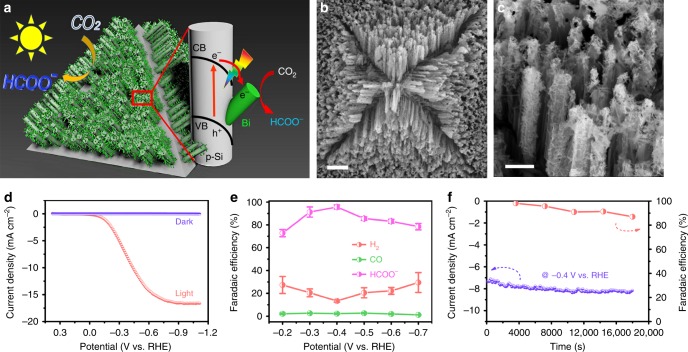
Photoelectrochemical carbon dioxide reduction on Si/Bi photocathode. **a** Schematic illustration of the structure of the Si/Bi photocathode and its working mechanism for PEC CO_2_RR. **b**, **c** SEM images of Bi_2_O_3_ nanotube (NT)-loaded Si nanowire array photocathode. **d** Polarization curves of Si/Bi under dark or 0.5 sun illumination. **e** Potential-dependent Faradic efficiencies for HCOO^−^, CO, and H_2_ from PEC. **f** Change of total current density and formate Faradaic efficiency at ‒0.4 V with time. Scale bar, 5 μm (**b**); 1 μm (**c**)

Encouraged by the superb CO_2_RR performance of NTD-Bi, we further investigated its potential as the co-catalyst to couple with Si photocathodes for photoelectrochemical (PEC) CO_2_RR, and pursued the solar conversion of CO_2_ to formate (Fig. 5a). To achieve this, hierarchical p-type Si nanowire arrays were fabricated and used as the light-absorbing semiconductor (see details in Methods). The nanowire array structure is designed to enhance the light harvesting capability, and improve its interaction with the co-catalyst^52–54^. Bi_2_O_3_ NTs were then introduced via spin-coating. Figure 5b, c are the SEM images of Bi_2_O_3_ NT-loaded Si nanowire array photocathode. Individual nanowires are ~5 μm in length and ~500 nm in diameter, uniformly distributed over the pyramidally textured substrate. Close examination reveals that their surfaces are decorated with fibrous Bi_2_O_3_ NTs (Fig. 5c). The intimate contact between the semiconductor and the co-catalyst would ensure the rapid transfer of photogenerated electrons across the interface.

PEC CO_2_RR was performed in CO_2_-saturated 0.5 M KHCO_3_ under the illumination of 50 mW cm^−2^ AM 1.5 G solar simulator (0.5 sun). Bare Si photocathode produces exclusively H_2_ (Supplementary Fig. 7). The introduction of co-catalyst markedly improves the PEC activity and shifts the selectivity. Since Bi_2_O_3_ NTs are cathodically converted to NTD-Bi prior to PEC CO_2_RR, the actual working co-catalyst is NTD-Bi. The photocathode is named as Si/Bi for the sake of clarity. No current is detected under dark (Fig. 5d). Its light polarization curve exhibits an onset potential at ~0 V, and a saturation photocurrent density of ~17 mA cm^−2^. The large saturation photocurrent density (close to the ideal value for Si photoelectrodes) suggests the full utilization of photogenerated electrons^55–58^. Product analysis discloses that the formate FE is maintained over 75% between approximately −0.7 and −0.3 V, and reaches the peak value of 96% at −0.4 V (Fig. 5e). A small amount of H_2_ and CO are detected as the side products. At last, the operation stability of Si/Bi was evaluated at −0.4 V for 5 h. The photocurrent density is maintained around 8 mA cm^−2^, and the corresponding formate FE only experiences a slight decrease to ~90% at the end of the evaluation (Fig. 5f). As far as we are aware, our photocathode demonstrates the most competitive PEC CO_2_RR performance in literature for formate production with balanced activity, selectivity and stability^59–62^.

## Discussion

In this study, we demonstrated that the CO_2_RR activity and selectivity of Bi were dramatically boosted by introducing surface defects. Bi_2_O_3_ NTs were first synthesized as the template via a facile solution method. They were featured with the unique double-walled tubular structure and highly fragmented outer surface. Under the cathodic polarization, Bi_2_O_3_ NTs were converted to Bi, while the tubular nanostructure was inherited. When tested using a standard H-type electrochemical cell, the converted electrocatalyst enabled CO_2_ reduction to formate with excellent selectivity (close to 100% over a broad potential window), great formate partial current density (60 mA cm^−2^ at −1.05 V), and impressive long-term stability (>48 h) in 0.5 M KHCO_3_, which were superior to all previous formate-producing electrocatalysts. When further tested on GDL using a flow cell reactor, NTD-Bi delivered even greater current density of 136 mA cm^−2^ at −0.86 V in 1 M KHCO_3_, and 288 mA cm^−2^ at −0.61 V in 1 M KOH, exceeding that of the commercialization requirement. *Operando* XAS measurements showed that NTD-Bi under the working condition contained abundant structural defects. DFT calculations indicated that the excellent activity and selectivity were due to the presence of defective Bi sites that stabilize the *OCHO intermediate. At last, NTD-Bi was integrated with p-type Si nanowire arrays for high-performance PEC CO_2_RR. Our study here unveils the tremendous potential of Bi in CO_2_RR electrocatalysis, as well as the remarkable impact of structural defects on its catalytic activity. It represents an important step forward to the commercialization of electrochemical CO_2_ utilization.

## Methods

### Preparation of bismuth oxide NTs

Totally, 100 mg of PVP (M.W. = 55,000 from Sigma-Aldrich) was first dissolved in 10 mL of ethylene glycol (≥99% from J&K Chemicals) and 0.1 mL of deionized water in a 50 mL three-necked flask at room temperature. 75 mg of bismuth acetate (99.99% from J&K Chemicals) was added to the above solution and formed a dispersion under the assistance of batch sonication for 30 min. Subsequently, the temperature of the reaction dispersion was rapidly raised to 195 °C, and maintained at this temperature for 15 min under magnetic stirring and N_2_ protection. The reaction was then quenched by adding 25 mL of ethanol and 10 mL of deionized water. Solid product was washed at least three times with absolute ethanol and deionized water, collected by centrifugation, and then lyophilized. Finally, possible organic residues on surface were removed by calcination in air at 300 °C for 1 h.

### Structural characterization

XRD was recorded on a PANalytical X-ray diffractometer. SEM images were taken on a Zeiss Ultra 55 scanning electron microscope operating at 15 kV. STEM imaging was conducted on a Nion HERMES-100 microscope under the accelerating voltage of 60 kV with a convergence semi-angle of 35 mrad. The low voltage setting was chosen to reduce irradiation damage to the sample. The BF and HAADF images were collected simultaneously using the BF and ADF detectors, respectively.

### Electrochemical measurements

A total of 1 mg of Bi_2_O_3_ NTs, 0.5 mg of Ketjenblack carbon, 10 μL of 5 wt% Nafion solution (Sigma-Aldrich) were first dispersed in 125 μL of water and 125 μL of ethanol under the assistance of ultrasonication for >30 min. The thus-formed catalyst ink was then dropcast onto a 1 × 1 cm^2^ carbon fiber paper (AvCarb P75 from Fuel Cell Store) to achieve an areal loading of 1 mg cm^−2^ as the working electrode. Electrochemical measurements were carried out in an H-type two-compartment electrochemical cell. The cathode compartment housed the working electrode and saturated calomel reference electrode (SCE); the anode compartment housed the graphite counter electrode. The two compartments were each filled with ~35 mL of 0.5 M KHCO_3_ electrolyte, and separated by a SELEMION anion exchange membrane. The electrolyte was bubbled with ultrapure CO_2_ gas for 1 h prior to measurements. The CO_2_ saturation was maintained during the measurements by continuously bubbling the electrolyte with CO_2_ at 20 sccm. All the potential values were measured against SCE and then converted to RHE. Polarization curves were recorded at 10 mV s^−1^. Reduction products were qualitatively and quantitatively analyzed using gas chromatography (Tianmei, GC7980) and ion chromatography (Dionex ICS-600) following the method described in our previous publications^25,63^. In brief, for gaseous products (CO or H_2_)

$${\mathrm{FE}}_{{\mathrm{gas}}}\left( \% \right) = \frac{{Q_{{\mathrm{gas}}}}}{{Q_{{\mathrm{total}}}}} \times {\mathrm{100\% }} = \frac{{\frac{v}{{{\mathrm{60}}\,{\mathrm{s}}/{\mathrm{min}}}} \times \frac{y}{{{\mathrm{24,000}}\,{\mathrm{cm}}^3{\mathrm{/mol}}}} \times N \times F}}{j} \times {\mathrm{100\% }}{,}$$

where *v* = 20 sccm is the CO_2_ flow rate, *y* is the measured product concentration in the GC sample loop, *N* = 2 is the number of electron transfer to form a molecule of CO or H_2_, *F* is the Faraday constant (96,500 C mol^−1^), and *j* is the total current. For formate in the catholyte

$${\mathrm{FE}}_{{\mathrm{HCOO}}^ - }\left( \% \right) = \frac{{Q_{{\mathrm{HCOO}}^ - }}}{{Q_{{\mathrm{total}}}}} \times {\mathrm{100\% }} = \frac{{n_{{\mathrm{HCOO}}^ - } \times N \times F}}{{j \times t}} \times {\mathrm{100\% }}{,}$$

where *n*_HCOO−_ is the amount of formate determined by the ion chromatography, and *t* is the electrolysis time.

### *Operando* XAS measurements

*Operando* XAS including XANES and EXAFS was performed at 5BM-D beamline of APS at Argonne National Laboratory using a home-made in situ XAS cell as described in our previous studies^64,65^. The working electrode was Bi_2_O_3_ NTs-loaded carbon fiber paper. A graphite rod and Ag/AgCl electrode were used as the counter electrode and reference electrode, respectively. The electrolyte was 0.5 M KHCO_3_. CO_2_ gas was bubbled constantly at 30 sccm during the measurements to maintain the saturated solution. Bi L fluorescence data were collected by a Vortex ME4 detector using the fluorescence mode. Data reduction and analysis were performed using Athena and Artemis software. For EXAFS fitting, Bi and Bi_2_O_3_ standards were used to obtain the amplitude reduction factor (*σ*_0_^2^) values, which were further used to calculated other fitting parameters^66^.

### Flow cell measurements

Flow cell measurements were performed in a custom-designed flow cell reactor made of polymethyl methacrylate plastic (Supplementary Fig. 4). It consisted of Bi_2_O_3_ NT-loaded GDL (1 mg cm^−2^, 4 × 4 cm^2^) as the cathode, a piece of anion exchange membrane (SELEMION, 4 × 4 cm^2^) as the separator, and a 20 wt% Ir/C-loaded GDL (1 mg cm^−2^, 4 × 4 cm^2^) as the anode. The cathode and anode compartments were each ~6.5 cm^3^ in volume. Ag/AgCl reference electrode with a diameter of 3.8 mm was located inside the cathode compartment. During the measurements, CO_2_ gas was directly fed to the cathode GDL at a rate of 80 sccm. The catholyte was 1 M KHCO_3_ or 1 M KOH. It was forced to continuously circulate through the cathode compartment at a rate of 10 sccm.

### Fabrication of Si/Bi photocathode and PEC measurements

Si nanowire arrays were prepared via the sequential anisotropic etching and isotropic etching of B-doped p-type Si wafers ((100)-oriented, 500 μm thick, 0.2–0.8 Ω cm^2^) following the method described in literature^67^. The wafer was then cut into small pieces of 1.5 × 1.5 cm^2^ in size, uniformly spin-coated with 600 μL of the alcoholic solution of Bi_2_O_3_ NTs (2 mg mL^−1^) and Nafion (0.1 wt%) at 500 rpm for 5 s and 1500 rpm for 20 s, and dried in air at 40 °C for 30 min. PEC CO_2_RR measurements were carried out following the procedure described in our previous publication^58^.

### Theoretical calculations

DFT computations were simulated with the Vienna ab initio Simulation Package^68^. The GGA function of the PBE form was used to describe the exchange-correlation energy^69^. After testing the energy cutoff and k-points convergence, we chose 460 eV as our cutoff energy, and used a 5 × 5 × 1 k-points grid to sample the unit cell Brillouin zone. We set a 15 Å vacuum space in the z-direction to avoid the periodic influence. The free-energy profiles were calculated by applying the computational electrode model^70^ to efficiently estimate the performance of electrocatalytic reactions. They were expressed as

$${\mathrm{\Delta }}G_{{\mathrm{ads}}} = {\mathrm{\Delta }}E_{{\mathrm{ads}}} + {\mathrm{\Delta }}E_{{\mathrm{ZPE}}} - T{\mathrm{\Delta }}S{,}$$

where Δ*G*_ads_ was the free energy of the adsorbates, Δ*E*_ads_ was adsorption energy of adsorbates, Δ*E*_ZPE_ and Δ*S* were the difference of *E*_*ZPE*_ and *S*, respectively, and *T* = 298.15 K was the temperature. *E*_*ZPE*_ and *S* were obtained by vibrational frequencies calculations with harmonic approximation and neglecting contributions from the slab. The contributions of relevant species to free energies are listed in Supplementary Table 2. The micro-kinetic simulations for the CO_2_ reduction processes were performed based on an established approach^50,51^. The modeling detail is described in Supplementary Note 1.

## Supplementary information

Supplementary Information

Source Data

## Data Availability

The data that support the findings of this study are available from the corresponding author upon reasonable request. The source data underlying Figs. 1a, 2a–h, 3b–d, 4b, c, 5d–f and Supplementary Figs. 1d, 2, 3a, 5c, 6a, b, 7a, b are provided as a Source Data file.
