# Supplementary material for: Structural defects on converted bismuth oxide nanotubes enable highly active electrocatalysis of carbon dioxide reduction
Source: Nat Commun. 2019 Jun 26;10:2807. doi: 10.1038/s41467-019-10819-4 (PMC6594929; doi:10.1038/s41467-019-10819-4)
Supplement: Supplementary file 1 — Supplementary Information [file 41467_2019_10819_MOESM1_ESM.pdf]

Supplementary Information

Structural defects on converted bismuth oxide nanotubes enable highly active electrocatalysis of carbon dioxide reduction

Gong *et al.*

1. Supplementary Figures

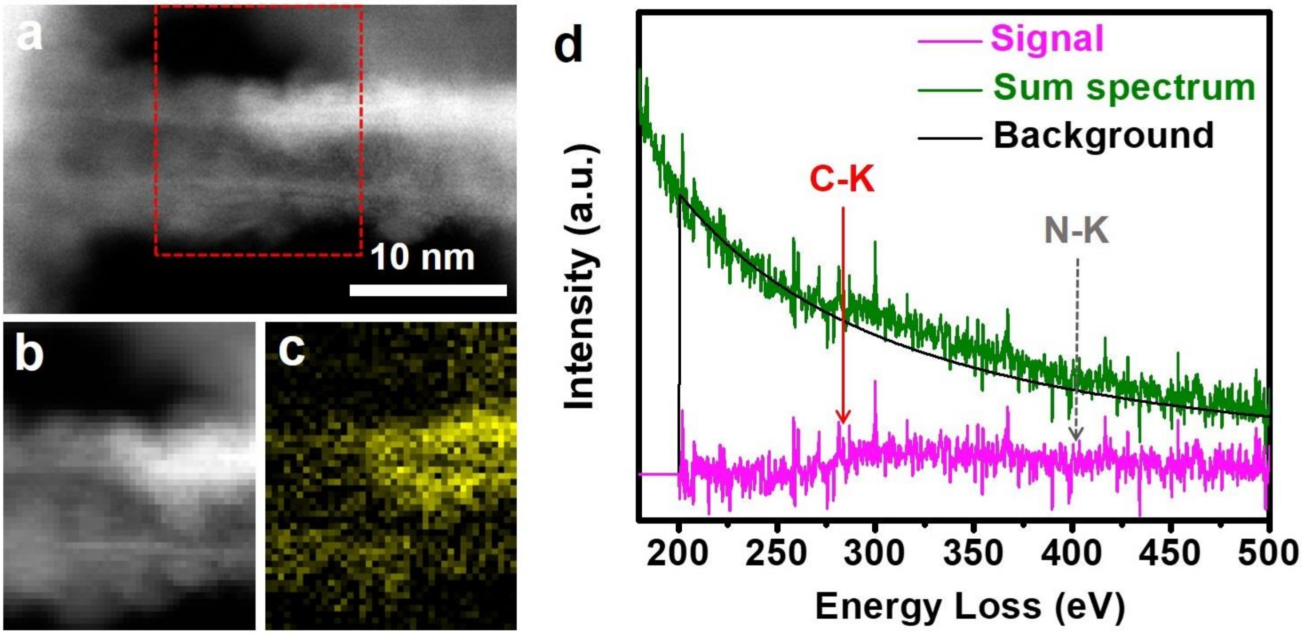

Supplementary Figure 1. (a) STEM-HAADF imaging of a Bi_2O_3 NT; the red dotted rectangle shows where the EELS mapping was acquired. (b) ADF image and (c) C-K edge mapping of the enclosed region in (a). (d) EELS spectrum in the C-K and N-K range.

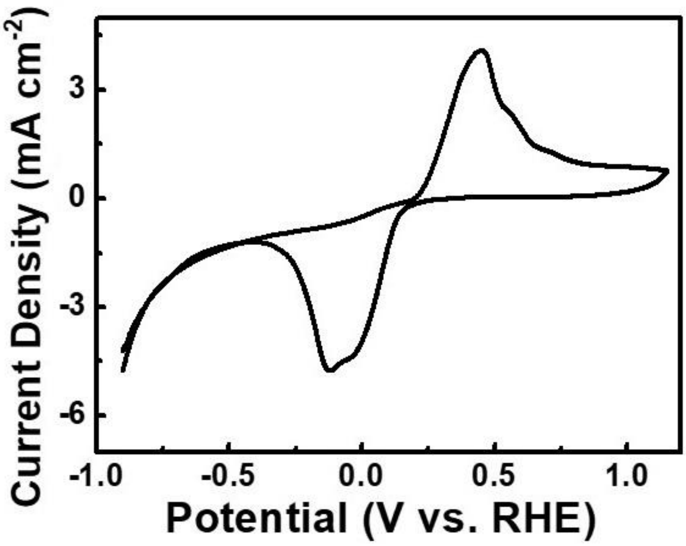

Supplementary Figure 2. CV curve of Bi_2O_3 NTs in N_2-saturated 0.5 M $KHCO_3$ showing the redox peaks assignable to the interconversion between Bi^{3+} and Bi^0.

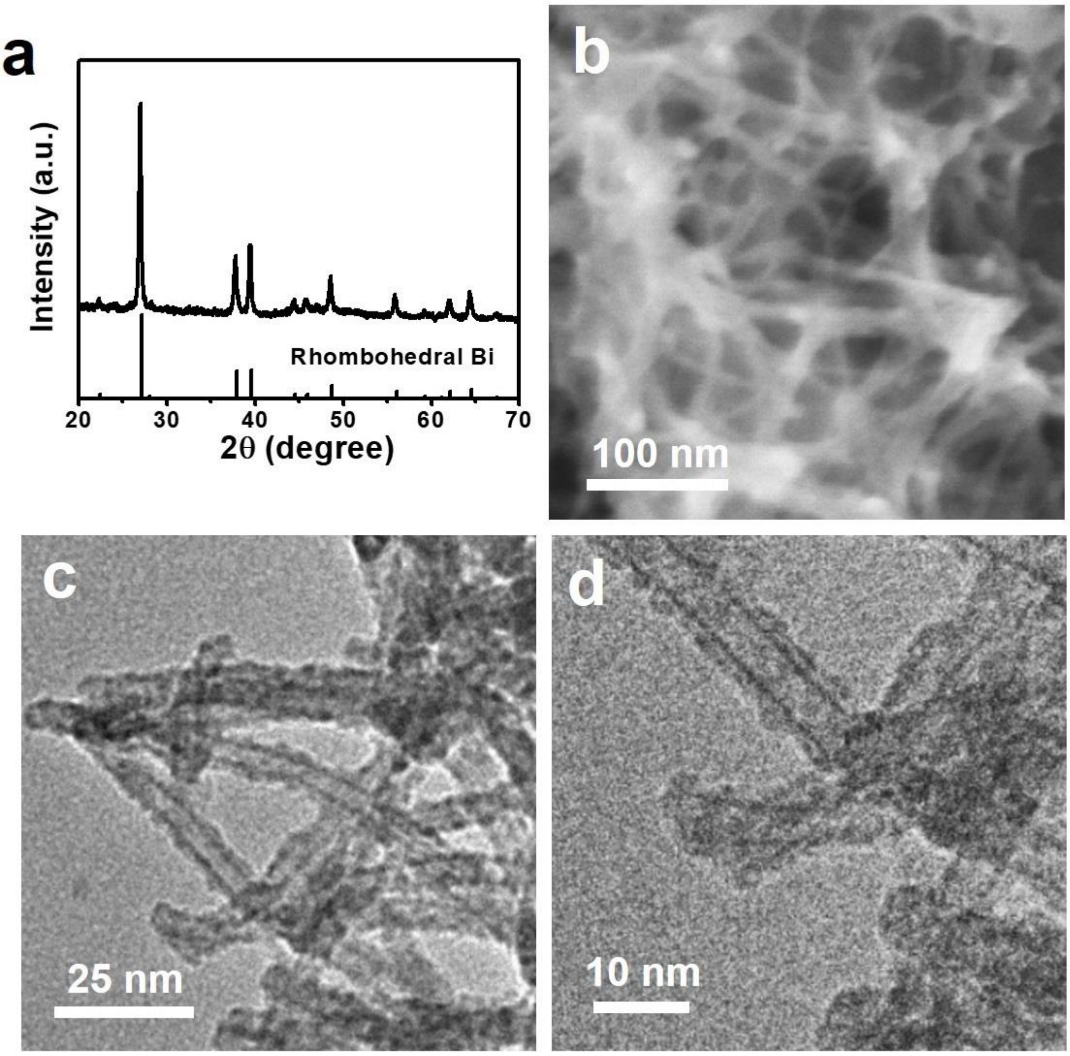

Supplementary Figure 3. (a) XRD pattern, (b) SEM image and (c,d) TEM images of NTD-Bi from the cathodic conversion of Bi_2O_3 NTs.

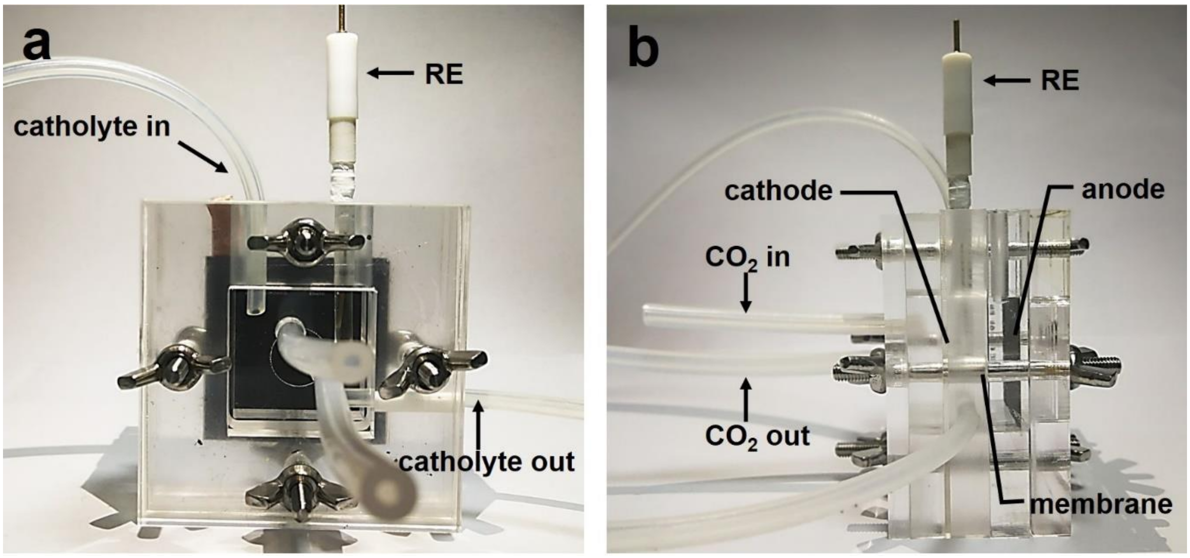

Supplementary Figure 4. Photos of our flow cell reactor viewed from (a) the front and (b) the side.

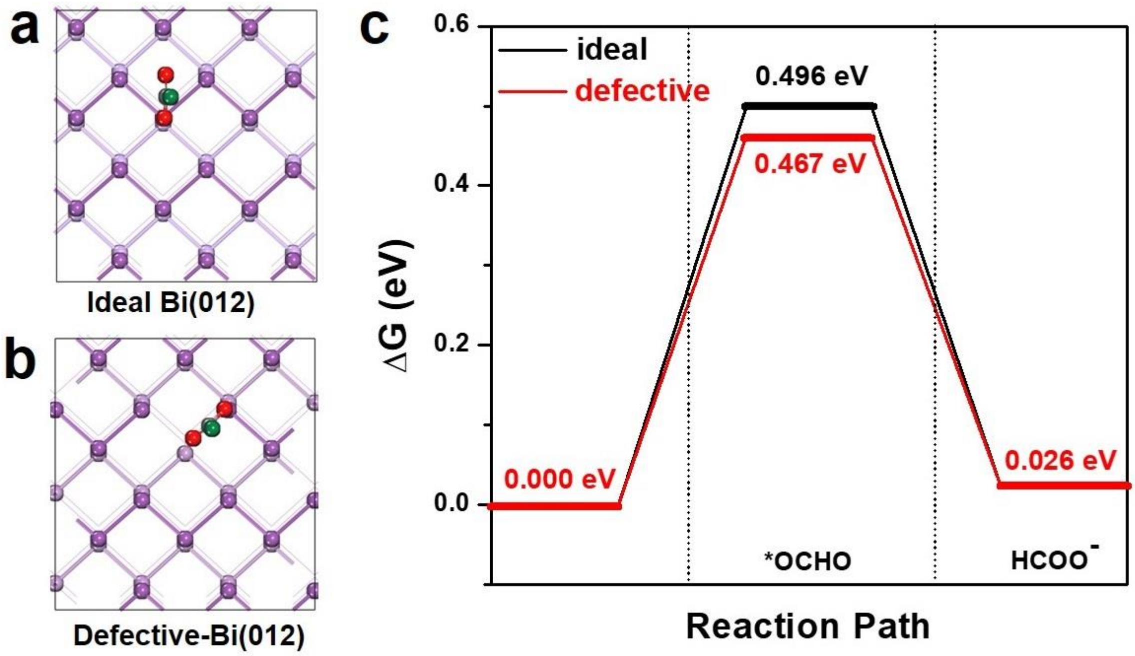

Supplementary Figure 5. Optimized geometry of *OCHO adsorption on (a) ideal and (b) defective Bi(012) planes; the dark purple atoms represent top layer Bi atoms, and the light purple represent Bi atoms in second layer. (c) Energy profile of the CO_2 reduction to $HCOO^-$ on ideal and defective Bi(012) surfaces.

Here, (012) plane was selected as the second example for DFT calculation because it has the most intense diffraction peak in the X-ray diffraction pattern (XRD) of Bi metal. On the ideal (012) plane, each Bi atom is connected with two equidistant nearest neighbor atoms in the same layer and one next nearest Bi in adjacent layer, forming a step like structure. DFT calculations show that the *OCHO immediate prefers to be adsorbed on the top site of Bi (Figure S5a). Its formation energy is +0.496 eV. To create structural defects, we remove one Bi atom from the top layer, break the initial wavelike structure and leave two coordinate unsaturated Bi atoms as active centers (Figure S5b). It is found that these two-fold coordinated Bi atoms stabilize *OCHO with ΔG =+0.467 eV (Figure S5c). This trend is generally consistent with our observation on Bi(001) that the presence of defects stabilize the key reaction intermediate and thereby enhance the reaction activity.

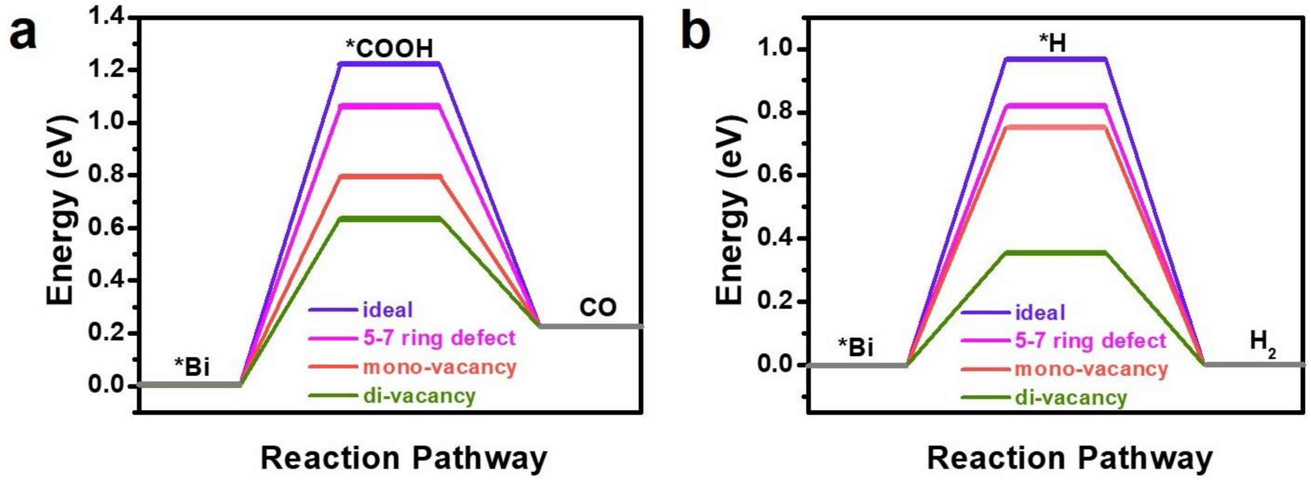

Supplementary Figure 6. Energy profile for (a) CO_2 reduction to CO (with *COOH as the reaction intermediate) and (b) hydrogen evolution reaction (with *H as the reaction intermediate) on ideal and defective Bi(001) surfaces.

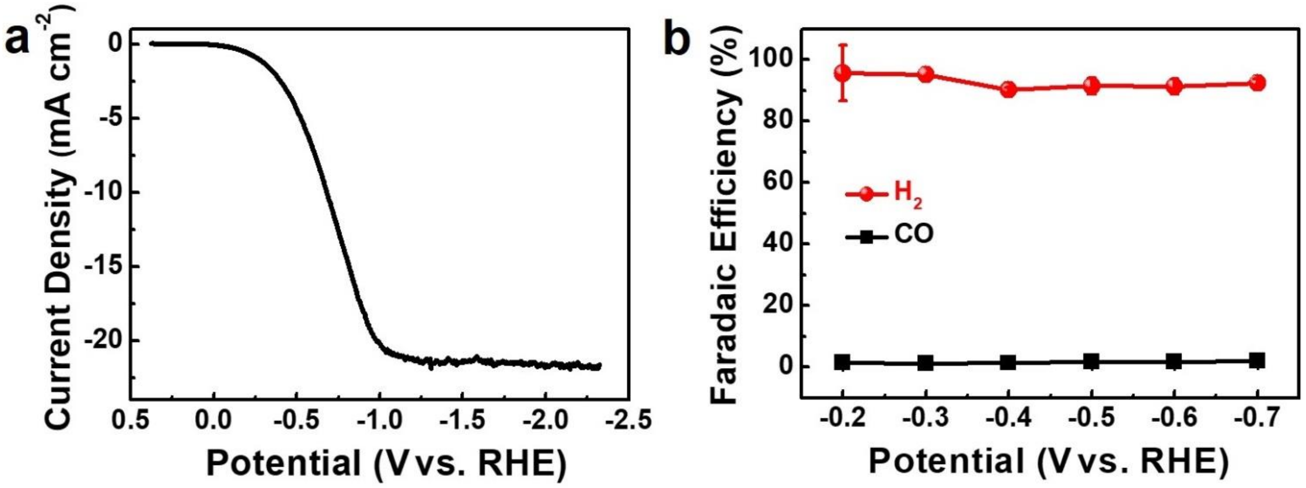

Supplementary Figure 7. (a) Polarization curve and (b) potential-dependent Faradaic efficiency for CO and H_2 on bare Si nanowire array photocathode in CO_2-saturated 0.5 M $KHCO_3$ under 0.5 sun illumination.

2. Supplementary Table

Supplementary Table 1. Fitting parameters of the EXAFS spectra of Bi catalyst at different potentials. R: distance; σ^2: mean-square disorder; E_0: energy shift.

	Scattering path	*CN*	*R* (Å)	E_0 (eV)	σ^2 (Å^2)
**Bi_2O_3**	Bi-O	1.0(2)	2.14(1)	-0.52(1.31)	0.0011(9)
	Bi-O	2.0(3)	2.22(1)	-0.52(1.31)	0.0096(8)
	Bi-O	1.0(2)	2.55(1)	-0.52(1.31)	0.0209(2)
	Bi-O	1.0(2)	2.65(1)	-0.52(1.31)	0.0202(5)
	Bi-Bi	2.0(3)	3.60(1)	-0.52(1.31)	0.0048(6)
**Bi**	Bi-Bi	3.0(4)	3.07(1)	-0.94(0.92)	0.0057(9)
	Bi-Bi	3.0(4)	3.53(2)	-0.94(0.92)	0.0189(9)
	Bi-Bi	6.0(8)	4.54(4)	-0.94(0.92)	0.0208(9)
	Bi-Bi	6.0(8)	4.74(5)	-0.94(0.92)	0.0219(7)
**OCV**	Bi-O	2.3(8)	2.27(4)	2.17(3.07)	0.0045(3)
	Bi-O	1.2(4)	2.60(4)	2.17(3.07)	0.0122(6)
**At -0.24 V**	Bi-O	1.1(3)	1.96(3)	-15.00(8.75)	0.0007(4)
	Bi-O	2.3(6)	2.19(4)	-15.00(8.75)	0.0062(6)
	Bi-O	0.9(6)	2.91(9)	2.89(3.86)	0.0055(3)
	Bi-O	0.9(6)	2.67(4)	2.89(3.86)	0.0012(3)
	Bi-Bi	2.6(1.8)	3.09(3)	2.89(3.86)	0.0079(1)
**At -0.78 V**	Bi-O	0.5(1)	2.59(2)	2.33(1.84)	0.0008(7)
	Bi-C	0.5(1)	3.02(5)	2.33(1.84)	0.0008(7)
	Bi-Bi	1.6(3)	3.10(2)	2.33(1.84)	0.0048(5)

The numbers in bracket are the last digit errors expect the red marked ones (full error).

Supplementary Table 2. Zero-pint energy correction (E_{ZPE}), entropy contribution (TS), and the total free energy correction (G–E_{elec}) for the studied system.

**Species**	E_{ZPE} **(eV)**	$-TS$ **(eV)**	G–E_{elec} **(eV)**
H_2	0.27	-0.41	-0.14
H_2O	0.56	-0.67	-0.11
CO_2	0.30	-0.67	-0.37
**HCOOH**	0.89	-0.89	0.00
**CO**	0.13	-0.61	-0.48
**H***	0.13	0.03	0.10
**COOH***	0.59	-0.26	0.33
**OCHO***	0.57	-0.31	0.26

3. Supplementary Note 1

The micro-kinetic modeling method utilized here for the formate partial current density calculation is based on the assumption that CO_2 adsorption, CO_2 protonation, *OCHO protonation and *HCOOH desorption are the main steps involved. The reactions can be expressed using the following steps:

$$CO_{2(aq)} \underset{k_{-1}}{\overset{k_1}{\rightleftarrows}} CO_{2(dl)} \tag{S1}$$

$$CO_{2(dl)} + H^+ + e^- + *_{Bi} \underset{k_{-2}}{\overset{k_2}{\rightleftarrows}} *OCHO \tag{S2}$$

$$*OCHO + H^+ + e^- + *_{Bi} \underset{k_{-3}}{\overset{k_3}{\rightleftarrows}} *HCOOH \tag{S3}$$

$$*HCOOH \underset{k_{-4}}{\overset{k_4}{\rightleftarrows}} HCOOH + *_{Bi} \tag{S4}$$

where $*_{Bi}$ represents the adsorption site, and $CO_{2(aq)}$ and $CO_{2(dl)}$ represent CO_2 in the electrolyte and the catalyst-electrolyte interface, respectively. Based on the above reduction steps, the rate equations of each species can be written as:

$$\frac{\partial x_{CO_2(dl)}}{\partial t} = k_1 x_{CO_2(aq)} - k_{-1} x_{CO_2(dl)} - k_2 x_{CO_2(dl)} \theta_{*_{Bi}} + k_{-2} \theta_{OCHO*_{Bi}} \tag{S5}$$

$$\frac{\partial \theta_{OCHO*_{Bi}}}{\partial t} = k_2 x_{CO_2(dl)} \theta_{*_{Bi}} - k_{-2} \theta_{OCHO*_{Bi}} - k_3 \theta_{OCHO*_{Bi}} \theta_{*_{Bi}} + k_{-3}\, \theta_{HCOOH*_{Bi}} \tag{S6}$$

These rate equations are solved numerically under steady state with the constraint from site conservation. For electrochemical step i, its equilibrium constant (K_i) can be expressed as:

$$K_i = A_i exp\left(\frac{\Delta G_i}{k_B T}\right) \tag{S7}$$

where ΔG_i is the free energy change of step i, and k_B is the Boltzmann constant. For non-electrochemical step i, the rate constant (k_i) is given by:

$$k_i = vexp\left(-\frac{\Delta E_b}{k_B T}\right) \tag{S8}$$

E_b is the HCOOH adsorption energy. In our work, we adopted the reported pre-exponential factors for both electrochemical steps ($A_i = 3.6 \times 10^4\ s^{-1}$) and non-electrochemical step ($\nu = 10^{13}\ s^{-1}$), which were derived from the fitting of experimental data of Au surface by Nørskov *et al.* (*J. Phys. Chem. Lett.* **4**,

388−392(2013)). While for electrochemical step, K_i is associated with the reaction potential (U vs. RHE), given by:

$$K_i = exp\left(-\frac{e(U - U_i)}{k_B T}\right) \tag{S9}$$

where U_i is the reversible potential of step i deduced by U_i = -ΔG_i/e. And the k_i is written as:

$$A_i \exp\left(-\frac{E_{\mathrm{a,i}}}{k_\mathrm{B} T}\right) \exp\left(-\frac{e\beta_i(U - U_i)}{k_\mathrm{B} T}\right) \tag{S10}$$
